# Situational Analysis of European and International Oral Health Policy Making for Quality Improvement

**DOI:** 10.1177/23800844251325540

**Published:** 2025-03-24

**Authors:** S. Akter, V. Fehrer, M. Lorenz, P. Jeurissen, S. Listl

**Affiliations:** 1 Heidelberg Institute of Global Health, Section for Oral Healthcare, Heidelberg University Hospital, Heidelberg University, Heidelberg, Germany; 2 Radboud University Medical Center, Radboud Institute of Health Sciences (RIHS), Department of Dentistry - Quality and Safety of Oral Healthcare, Nijmegen, the Netherlands; 3 IQ Healthcare, Radboud University Medical Center, Nijmegen, The Netherlands

**Keywords:** dental care, health care quality, health care disparities, health services needs and demand, policy, Europe, qualitative research

## Abstract

**Introduction:**

Despite increasing dental expenditures, the burden of oral diseases has not decreased. The room for improving the quality of oral health care (OH) remains large. The purpose of this study was to explore the current understanding, needs, efforts, and actions in European and global policy making for oral health quality improvement.

**Methods:**

Drawing from qualitative methodology comprising desk research and semi-structured interviews, a situational analysis was carried out. Interviews with experts in international oral health policy were recorded, transcribed, and analyzed inductively and iteratively.

**Results:**

The interviews with 13 participants representing 11 organizations provided diverse insights into policy making for improving the quality of OH. Thematic analyses identified 4 main themes: (1) perception and understanding of quality improvement (QI) in OH policy making, (2) prioritization of QI, (3) efforts and actions for QI, and (4) stakeholder engagement. Three maps were also generated: situational map, social world map, and positional map. Participants acknowledged several facilitators and barriers and provided QI ideas but also expressed concerns. They said that QI was underserved and not properly prioritized. Competing goals and financial limitations were considered major barriers for QI. Some organizations described that they are involved in OH QI and took various initiatives to improve quality, whereas others acknowledged that QI efforts could be expanded. Participants also expressed a necessity for better coordination among stakeholders and joint action on QI to enhance the overall OH of the population in Europe and globally.

**Conclusions:**

The findings of this study suggest that there is substantial room for improvement in European and global policy making concerning the QI of OH. While stakeholders seem to recognize the relevance of OH QI, competing priorities and limited resources seem to be perceived as barriers to scaling up QI efforts. The potential of international synergies in QI for OH is emphasized.

**Knowledge Transfer Statement:**

The findings of this study provide valuable insights for decision makers and stakeholders who aim to improve oral health care policy making to optimize oral health care in Europe and beyond by offering a deeper understanding of the current situation of international quality improvement efforts for oral health care.

## Introduction

Oral health remains a major public health concern, even though most oral diseases are preventable (Susarla et al. 2022) and expenditure on dentistry has increased over the past several decades (Jevdjevic et al. 2021). The global burden of oral diseases has not decreased (World Health Organization [WHO] 2022), and oral health care (OH) has improved only slowly (Agency for Healthcare Research and Quality 2022), as improving access and quality of oral health remains a global challenge (Kassebaum et al. 2017; Peres et al. 2019; Susarla et al. 2022). In addition, there are significant disparities in access to care and treatment costs across Europe (Winkelmann, Gómez Rossi, Schwendicke, et al. 2022). Oral health policies are defined as formal statements or courses of action by institutions, organizations, services, funding arrangements, groups, and individuals that provide general guidance and indicate future directions for improving oral health (Strippel 2008). Despite the high disease burden, oral health is often overlooked in public health policy (WHO 2022). In many countries, including several European countries, oral health is not adequately addressed in health policies and strategies (Sloth-Lisbjerg 2023). Inadequate information and surveillance systems combined with a low prioritization of public oral health research are the main bottlenecks to developing more effective oral health policies (WHO 2022). Therefore, improving the quality of oral health is an important public health issue that requires the attention of policy makers at both the European and global levels and encompasses several dimensions, including patient safety, effectiveness, efficiency, patient centeredness, equity, timeliness, and access to care, as described in the international working definition of oral health quality improvement (QI) (Righolt et al. 2020). Effective national and international policies and measures are needed to promote oral health and prevent disease (Petersen et al. 2020).

Previous research has explored various aspects of OH policy development, including the influence of stakeholder engagement (Chazin and Center for Health Care Strategies 2015), the role of evidence-informed decision-making (Listl et al. 2023), and the challenges of implementing effective interventions (Simpson 2011). Funding mechanisms and capacity-building initiatives are essential to ensure the long-term sustainability and successful implementation of OH policy (Fisher et al. 2023). However, the lack of data on OH and the effectiveness of different interventions make it difficult to make informed policy decisions (National Institutes of Health 2021).

To improve the quality of OH at the European and global level, future policies and actions need to consider these difficulties and the current state of OH policy. Nevertheless, a comprehensive perception of the current state of understanding, needs, efforts, and actions in the context of European and global policy making to raise the standard of OH remains fragmentary. This hinders the development and implementation of effective interventions to improve the quality of OH. To this end, the European Union (EU) project DELIVER (DELiberative ImproVEment of oRal care quality) was designed to improve the quality of OH through deliberative dialogue and action involving citizens, patients, providers, payers, and policy makers (Listl et al. 2024). To identify existing challenges and opportunities for QI and in the absence of previous such work, situational analysis (SitA) forms a central element of the DELIVER project.

The present study aims to address this knowledge gap by exploring the current understanding, needs, efforts, and actions in the context of European and global policy making for improving OH quality. Considering the previous absence of such evidence, the present study adds novel and meaningful evidence to the knowledge base on QI for OH.

## Methodology

### Study Design

This qualitative study was conducted using SitA methods and used semi-structured interviews to look into participants’ understanding, efforts, and actions concerning European and international policy making for enhancing OH quality. A 17-item semi-structured interview guide was developed based on the Consolidated Framework for Implementation Research (Damschroder et al. 2022), pretested with the researchers (S.A. and V.F.), and finalized. The Medical Faculty Heidelberg Ethics Committee reviewed and approved the study (reference: S-089/2023). The methods and findings of the study were presented using the Standards for Reporting Qualitative Research checklist as a guide.

### Desk Research

Desk research was the first step to identify key European and international (oral) health organizations. Further information was collected by looking through the organisations websites and at gray literature, such as statement papers or other publicly available information published by the organizations. This initial step provided valuable information about how various actors are connected and how they represent themselves to the public. With this information, the researchers started the mapping process of the SitA.

### Qualitative Interviews

By conducting qualitative interviews, we aimed to enrich the findings from our desk research and gain a broader perspective on participants’ professional and personal experiences of QI in OH.

### Participant Recruitment

A first brainstorming session was held to identify big actors in OH based on the researchers knowledge, previous experience, and desk research. The researchers also searched scientific journals to find organizations, institutes, or authors. Input was also gathered from the DELIVER consortium concerning recommendations on organizations to contact. An initial list of 42 European and global organizations has been developed. Most interviewees were recruited through personal contacts of the DELIVER consortium and interviewees suggesting other potential interviewees, as the response rate was very low for the organizations that were approached via the publicly available email addresses on the respective websites. The invitation and recruitment process were improved along the way; hence, the response rate was improved. Finally, 13 participants were recruited for this study through purposive and snowball sampling. After conducting 10 interviews, data saturation was discussed and preliminarily achieved. Three further interviews were conducted to promote this finding. It was considered that additional interviews would not result in better insight, and an in-depth understanding of the research question was believed to have been gained.

### Data Collection

Two researchers (S.A. and V.F.) performed the interviews virtually via video call using the platforms Webex and Zoom. All interviews were audio-recorded, and researchers took field notes throughout the interviews. All participants received the information leaflet and consent form prior to the interviews, and informed written consent was gained. All interviews were transcribed verbatim using the GoSpeech transcript software and then proofread by both researchers to ensure accuracy. Before the transcription, all personal information about the participants was removed from each transcript, and the file was saved pseudonymized. All audio recordings and original transcripts were securely stored on the University Hospital Heidelberg servers.

### Data Analysis

All transcripts were analyzed using the MAXQDA Analytics Pro 2022 software. S.A. and V.F. coded each of the transcripts. This study was conducted based on the SitA method, which is developed from the grounded theory approach, to generate explicit maps of contextual situations inductively and iteratively (Clarke et al. 2018). Thematic analysis was used to identify and describe the main themes (Braun and Clarke 2012). The codebook included inductive content-driven codes derived from the interviews. The analysis started with line-by-line coding. This involved reviewing and verifying the transcripts by S.A. and V.F. to identify relevant concepts, giving each idea a code name, and listing and defining codes in a codebook. Coded transcripts were cross-checked to assess intercoder agreement at regular intervals using MAXQDA. After checking the intercoder agreement each time, S.A. and V.F. met and discussed the codebook to resolve discrepancies. As a final step, discussions were conducted by S.A. and V.F., and the intercoder agreement was increased to 72.38%.

Themes were identified, revised, and edited throughout the analysis process. Following the SitA approach, the 2 researchers revisited the maps iteratively. The researchers S.A. and V.F. started creating the maps during the desk research. They started with a messy map based on desk research and meetings with colleagues regarding major oral and general health care players and their efforts. Once they gathered more information in that field, an ordered and relational map was produced to demonstrate the similarities and overlaps between the various organizations discovered during the desk research. The maps were finalized after conducting all interviews and analysis. Three maps were generated: a situational map, a social worlds and arenas map, and a positional map (Clarke et al. 2018). While the situational map aimed to present essential contextual elements of OH QI in Europe and globally, the social worlds/arenas map introduced groups of organizations defined by their common interests or concerns. The positional map illustrates the positions taken within the major discursive issues that emerge in the data. It highlights the range of positions on topics that are not associated with individuals or organizations but with the positions in the discourses as reflected in the data (Martin et al. 2016).

### Researcher Reflexivity

The 2 researchers practiced reflexivity to analyze and improve validity. Intercoder reliability was evaluated, which promotes reflexivity (O’Connor 2020). To ensure trustworthiness, the researchers sought feedback from academic colleagues, conducted iterative data analysis, and provided a detailed and thorough description of their study process and methods (Nowell et al. 2017). The study’s trustworthiness was further strengthened by using multiple data sources (interviews and literature reviews) and involving researchers with different backgrounds (health services researcher and dentist) (Nowell et al. 2017).

## Results

### Participant Characteristics

Thirteen semi-structured interviews were conducted between March 31 and April 14, 2023. Thirteen participants represented 11 different organizations, 7 international and 4 European organizations. Two of the organizations were represented by 2 participants each. Table 1 summarizes the sociodemographic data of the study population. Participants were diverse in their professional backgrounds and predominantly male. The interview duration ranged from 25 to 52 min, averaging 38.5 min. Table 1 is divided according to the type of organization to which the participants belong.

**Table 1.** Sociodemographic Data of the Study Population.

Type of Organization	EU or Global	Position	Sex
Professional (3)	EU (1) Global (2)	President (2) Secretary (1)	Male (3)
Advisory (2)	EU (1) Global (1)	Consultant (1) Member (2)	Male (2) Female (1)
Joint initiative (2)	EU (2)	Chairman (1) Coordinator (1)	Male (2)
Commission (1)	Global (1)	Member (2)	Male (1) Female (1)
Student (1)	Global (1)	President (1)	Female (1)
Consortium (1)	Global (1)	Chairman (1)	Male (1)
Charity (1)	Global (1)	Chairman (1)	Male (1)
*n* = 11	*n* = 11	*n* = 13	*n* = 13

EU, European Union.

### Qualitative Findings

**Figure 1.**
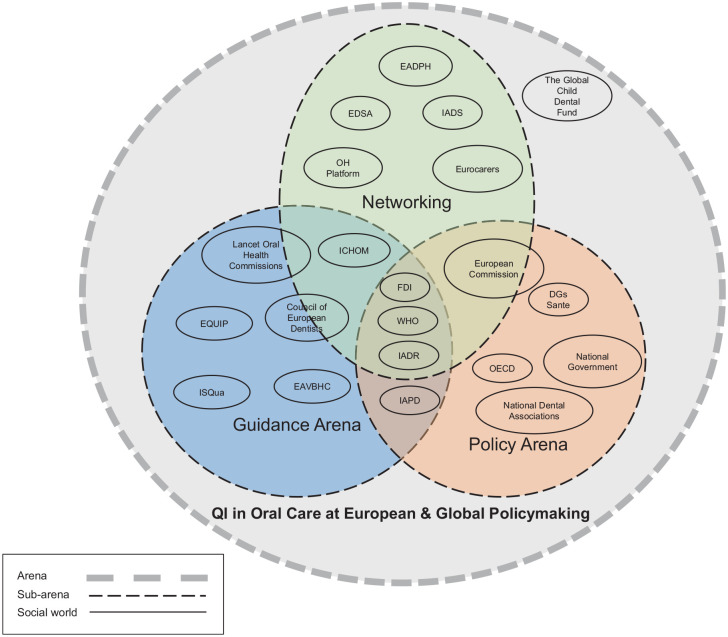
Social worlds/arenas map. The figure shows the social worlds or arenas for improving the quality of dental care at the EU and global policy levels. CED, Council of European Dentists; DGs Sante, Directorate-General for Health and Food Safety; EADPH, European Association of Dental Public Health; EDSA, European Dental Students’ Association; EQUIP, European Association for Quality and Patient Safety in General Practice; EAVBHC, European Association of Value-Based Healthcare; FDI, World Dental Federation; IADS, International Association of Dental Students; ICHOM, International Consortium for Health Outcomes Measurement; IADR, International Association for Dental Research; ISQua, International Society for Quality in Healthcare; IAPD, International Association of Paediatric Dentistry; OH Platform, Oral Health Platform; OECD, Organization for Economic Cooperation and Development; QI, quality improvement; WHO, World Health Organization.

**Figure 2.**
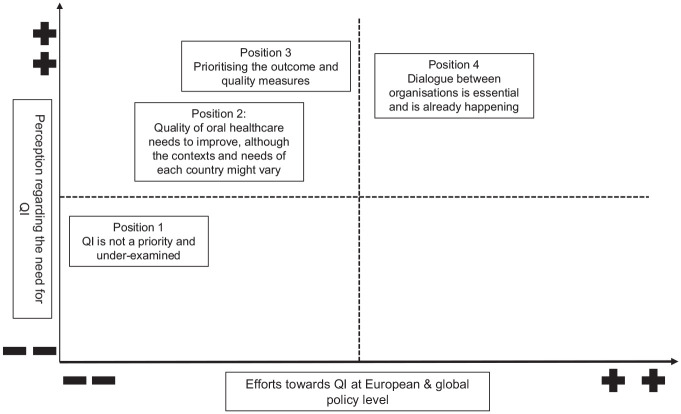
Positional map showing different positions within important discursive issues that appear in the data.

Four main themes and 3 maps were generated from the data. The 4 identified main themes were (1) perception and understanding of QI in OH policy making, (2) prioritization of QI, (3) efforts and actions for QI, and (4) stakeholder engagement. The 3 maps are a situational map (Table 2), a social worlds/arenas map (Fig. 1), and a positional map (Fig. 2).

**Table 2.** Situational Map Highlighting the Most Prominent Components of Oral Health Care Quality Improvement in the European Union and Globally.

Headline	Content
Individual human elements/actors	President, chairman, consultant, secretary, chief dental officer, coordinator
Collective human elements/actors	International organizations, EU organizations, government, national organizations including dental associations and professions, industry, patient organizations
Concepts/ideas/commitments	Digital dental instruments and materials, digitalization of dentistry, public health approach, monitoring, OH advocacy
Implicated/silent actors/actants	Funding, lobbying, sponsoring, corporations, brands
Elements related to resources/economics	Wish for UHC and global standardization of OH, financial crisis and lack of funding, lack of workforce and data, QI is not a priority, no common goals at EU level, focus on cancer research
Sociocultural/symbolic elements	Inequality in access to care, social barriers, cultural variations, education, training, medical deserts
Temporal element	COVID-19 pandemic
Spatial elements	Countries within the EU level vary significantly and even more globally
Major issues/debates	WHO OH report, WHO action plan, EU policies, fluoride policies, PaRIS project (patient-reported indicator surveys), BeWell EU project, UHC, Childsmile project in Scotland
Related discourses (historical, narrative, and/or visual)	Quality needs to be improved, more outcome oriented, not disease oriented, focus more on prevention than intervention, focus more on patient outcomes, different approaches need to be taken, timeliness of care, and continuing care
Communication paths/ dialogues	Policies and guidelines, passing of laws, publishing papers, statements, and positions, congresses, conferences and meetings, stakeholder engagement, intersectoral approach

EU, European Union; OH, oral health care; QI, quality improvement; UHC, universal health coverage; WHO, World Health Organization.

#### Theme 1: Perception and understanding of QI in OH policy making

Participants exhibited a good understanding of QI and identified elements that contribute to the quality of OH, including effectiveness, patient centeredness, quality control, quality of life, timeliness, accessibility, efficiency, safety, and training. To achieve optimal OH, most participants emphasized the importance of evidence-based interventions and patient-centered attributes of accessibility, safety, timeliness, and quality of life.

You know, it has to have certain characteristics, and you can be defensive to it. So, you can say it needs to be safe. But that’s not enough for me. It’s also these other characteristics, particularly those that are evidence for effectiveness and efficiency of care, but also acceptability to the recipients. (INT_6, Pos. 18)

OH providers must be well-educated and trained to perform the appropriate treatment. Quality control protocols are also crucial in enhancing the quality of care. The lack of training and quality control may negatively affect patients, and a lack of quality control exists, as mentioned by one participant.

But in most European countries, not all, or at least in my own country, there is no quality control to see from the outside. (INT_3, Pos. 10)

With regard to the global optimization of QI, the recognition of the different needs and contexts of different countries and the exchange of knowledge between nations was emphasized. A strong need to learn from best practices, share knowledge, and connect people from different countries emerged as an essential concept. Some participants pointed out the importance of universal goals, such as international standards for patient outcomes, universal health coverage (UHC), international collaboration, and standardization. Others stressed the need for vigorous monitoring and outcome-oriented care.

In OH, we should have some universal goals, like reducing dental caries. Then, we should be able to monitor our progress towards that. And that’s how we would measure quality. And then, in another dimension, perhaps internationally, we would look at what best practices are in different countries to see if there are other things we should be doing. But all the time, I think it’s very important that the context of each country or region could be very different from another. So it’s what is appropriate for a certain place. (INT_2, Pos. 10)

Participants perceptions of QI at the European level varied. While acknowledging some EU-level initiatives like the adaptation of the WHO action plan and specific initiatives on reducing oral cancer and phasing down amalgam, concerns remained about a lack of Europe-wide common framework and goals for OH QI.

So, from that point of view, you could say we’re not working yet towards a particular goal. Now, I suppose if the EU and European countries adopt the WHO action plan, that’s probably the closest thing we will have to a set of goals or, you know, quality, that we will aim towards [. . .] reduction in disease (INT_2, Pos. 14)

A key finding was the limited awareness of existing QI initiatives, policies, and guidelines influencing OH at international and European levels. Many commented that they were unfamiliar with ongoing global and Europe-wide QI dialogues and initiatives.

Again, because I don’t know much about what’s going on internationally now, it’s hard for me to comment on how to improve it or make changes. (INT_1, Pos. 38)

The information gathered from the interviews indicated that participants had diverse perceptions and understanding of QI in OH policy within and outside of Europe, which is crucial for effective prioritization.

#### Theme 2: Prioritization of QI

The qualitative analysis revealed an overarching theme about the prioritization of QI. Almost all participants acknowledged the necessity of enhancing quality and implementing QI initiatives in OH. Some ideas have been proposed, such as developing a Europe-wide framework for improving quality and member states setting realistic targets to improve this field. Meanwhile, participants discussed the potential barriers and drivers of OH QI that they could identify.

So, I think it’s important that we don’t set up ideals, that member states can set their own targets and that we have realistic targets for how we move forward in health improvement. I think it’s good to have a core standard. That doesn’t mean that people can’t go beyond the core standard. (INT_9, Pos. 98)

As stated in the interviews, global standardization of OH can potentially drive QI by reducing disparity. Focusing more on preventive care than intervention and having a QI framework and common goals are also recognized as facilitators for QI in European and global OH policy making.

So, instead of just providing the treatments, we’re also focusing on prevention. (INT_7, Pos. 34)

One of the most frequently mentioned statements was main barriers to improving quality, which impedes policy making. The most prevalent barriers were access to care, lack of money and funding, knowledge and workforce for QI, cultural differences, and disease-oriented care. Participants reported that working on QI is also challenging due to leadership and governance issues as well as a lack of regulatory bodies and data.

You look at financial access and geographical access: where in the countries are these services, and where are they available, and cultural access? (INT_10, Pos. 170)

The cost of OH can lead to financial ruin for individuals who are uninsured or underinsured, as they are required to pay out of pocket for these expenses. Unfortunately, access to dental care is often deprived by income inequality, as some people have access to dental services while others do not. This inequality can result in catastrophic OH expenditures that leave individuals living in poverty.

And oftentimes, it’s also a reason for catastrophic health expenditure pushing people into poverty because they have a complicated dental problem that they need to pay privately for. (INT_11, Pos. 42)

Overall, while participants recognized different facilitators and barriers and proposed ideas for QI, they also expressed their concerns. They said QI was underserved and not appropriately prioritized. Competing goals and financial limitations were considered to push QI further down the agenda.

#### Theme 3: Efforts and actions for QI

The participants demonstrated that their organizations undertook some efforts and actions to promote OH QI across Europe and the globe. These efforts include bringing leaders, researchers, and member states together, supporting QI projects, collecting standardized data, emphasizing research, and knowledge sharing. Some organizations host conferences and workshops to bring together leaders and researchers from different countries. In contrast, others create shared platforms to facilitate knowledge exchange, foster research collaboration, and strengthen the foundation for informed policy decisions. Bringing the member states and researchers together is one of the main goals of a few organizations. It could create more opportunities for QI in OH in Europe and globally and allow them to work more closely together.

We have brought together over 1000 clinical leaders and patients from over 60 nations to discuss and agree on what is measured disease by disease. (INT_8, Pos. 2)

It is a facilitation group for our member states and enables member states to work more closely together. (INT_9, Pos. 10)

Few organizations actively support ongoing QI initiatives by providing technical assistance, resources, and expertise, while others support indirectly through the respective national dental associations. These direct or indirect supports allow individual countries to adopt best practices and tailor QI efforts to their specific needs.

We support them, but we don’t do it ourselves. (INT_3, Pos. 90)

Emphasizing standardized data collection, analysis, and research is one of the main focus areas for some organizations. They collect the data to develop evidence-based interventions and solutions and study the dental workforce and financial aspects to guide future QI strategies. However, some participants noted that the collection and standardization of data are difficult to achieve, even in collaborative systems such as the EU.

On the one hand, there are statistics on the structural side, so we try to capture the overall statistics on the workforce. What’s the dentist workforce per 100,000 people? My colleagues are very interested in the financial side, especially whether the part of dentistry is covered by public finance. (INT_10, Pos. 18)

Openly sharing knowledge and best practices across countries is considered very important. This cooperative strategy ensures further dissemination of effective QI strategies and speeds up global progress toward equitable OH.

It allows us to identify best practices and, therefore, learn from experience and learn from the differences between practices. (INT_8, Pos. 126)

Some of the most common goals were to provide equitable access to OH, improve the quality of OH, and establish a scientific community to create ideas and define QI standards. Several other organisations goals were revealed throughout the interviews, including raising worldwide awareness of OH, achieving UHC, and increasing quality and outcomes.

I’m trying to say that the role is to improve and promote good OH and reduce inequalities, so the quality of OH goes through this. (INT_6, Pos. 58)

The participants referred to the following QI activities and ongoing projects: the WHO global oral health status report, the WHO strategy of action plan, the Paris project for primary care in Europe, the BeWell EU project, fluoride policies, EU policies, and one national-level project called the Childsmile program in Scotland. They also listed the names of a few organizations that have influenced the external policies of QI, including WHO, the World Dental Federation (FDI), the EU Commission, the Organization for Economic Cooperation and Development, International Association for Dental Research (IADR), and International Consortium for Health Outcomes Measurement (ICHOM).

You then have, you know, bodies like the FDI and IADR. For example, IADR has policies that support Minamata and amalgam removal. And so you have IADR, you have FDI, and then you would have the various professional organizations (INT_2, Pos. 38)

OECD has been quite influential. They have worked on quality measurement and quality benchmarking. (INT_8, Pos. 82)

Participants mentioned past QI efforts with specific initiatives, including research as an objective in the WHO action plan through lobbying and advocacy, ensuring that oral cancer is included in the general cancer action plan, and prohibiting amorphous nanoparticle silica in toothpaste. While discussing previous projects, participants also proposed ideas such as digitization, which, if properly implemented, might improve the quality of OH in the future. In addition, the organization’s participants said that they accept and assess new ideas and try to find new ways to solve the problems in OH with evidence-based solutions.

So, we had some success, for example, where the commission last year, I think, was going to ban the use of this amorphous nanoparticle silica in toothpaste that, you know, the platform was very good at that (INT_2, Pos. 54)

We embrace new ideas by listening to other member states and their initiatives and looking at evidence. (INT_9, Pos. 94)

Although some of the organization’s participants have already taken many actions and efforts at European and global levels, other organizations are not that active. It was also frequently mentioned that what the organizations are not doing or what they are doing is insufficient. On the other hand, some said that QI is out of their scope entirely and their organization is not responsible for improving or managing quality.

In my opinion, we are not doing enough. (INT_1, Pos. 42)

We follow the legislation. . . . We don’t do any service related to quality management. That’s not our purpose. (INT_3, Pos. 26)

However, some organizations generally described themselves as being heavily involved in OH QI and taking different initiatives to improve the quality, while others acknowledged that QI efforts could be expanded.

#### Theme 4: Stakeholder engagement

Most participants acknowledged the need for stakeholder engagement and dialogue in achieving meaningful QI in OH across Europe and globally. A wide range of key players who could expedite progress in OH QI policy making were identified. International organizations comprising the WHO, FDI, ICHOM, International Association of Paediatric Dentistry, and International Society for Quality in Healthcare have been recognized as significant actors in global leadership and QI frameworks that foster OH. The EU Commission, Eurocarers, European Association for Quality and Patient Safety in General Practice, Directorate-General for Health and Food Safety, and Chief Dental Officers have been specified as leading actors in Europe. Governments, national dental associations, private industries, and patient organizations are key stakeholders and have influencing power. Approaching QI from an intersectoral perspective is recommended. Collaboration among professionals is essential for enhancing OH and raising the standard. This approach also facilitates engagement between public and private sectors.

All of the stakeholders are important, and WHO has laid it out. You have government, international organisations, and national organisations like dental associations. And I think you have your industry to be in it as well. Those are the important players because I don’t see how we get real progress without all those people being involved. You know, governments are absolutely essential. (INT_2, Pos. 34)

In addition, 1 participant shared that in the EU it is often difficult to achieve seamless collaboration between sectors, which prevents them from implementing QI successfully, whereas the United States has a more integrated approach.

I could only sit there and be a little bit envious that in the US, you have the funders, the research people, and the professionals, not doing everything together but willing to sit down together. They overlap and move together much more coherently than in Europe on specific issues like what we’ve been talking about. (INT_2, Pos. 70)

Dialogue between countries and organizations is considered an essential element for QI, and this can bring different stakeholder groups together and help them exchange knowledge and best practice examples. It was stated that some dialogue is already happening between organizations and countries, but more dialogue should happen between stakeholders in the future. It was also highlighted that there is already a broad-based advocacy group in the EU that promotes OH improvement in addition to contributing to dialogues.

There are big congresses all over Europe every year attracting people from all the neighbour countries. There are also places where people meet and discuss the best possible treatment with good clinical examples. So, the World Dental Association, FDI, organises a big event every year in some parts of the world. The American Dental Association, [and] my own dental association [are] also doing that (INT_3, Pos. 62)

Overarching opinions expressed particularly a need for better collaboration among stakeholders and cooperative action on QI projects to benefit the overall OH of the people in Europe and globally. To fulfil this aim, stakeholders must prioritize common goals, communicate frequently, and build partnerships.

### Situational Maps

The researchers S.A. and V.F. started with a messy map based on desk research and meetings with colleagues regarding major oral and general health care players and their efforts. Once they gathered more information in that field, an ordered and relational map was produced to demonstrate the similarities and overlaps between the various organizations discovered during the desk research. Following the SitA approach, the 2 researchers revisited the maps iteratively, and the final 3 maps were created: a situational map (Table 2), a social worlds/arenas map (Fig. 1), and a positional map (Fig. 2).

Twelve headings were proposed by Clarke for the situational map, but she recommended that researchers should adapt the headings that are appropriate for understanding their data (Clarke 2003). Based on interviews, notes, and desk research, the situational map (Table 2) highlights the most prominent components of OH QI in Europe and globally.

Two researchers laid out all relevant components by asking who and what matters in this situation and what elements make a difference in this situation (Clarke et al. 2018). For instance, the concepts/ideas/commitments and elements related to resources/economics categories were developed by the researchers based on the data. Individual and collective human actors who play a vital role in QI, such as organizations and their leaders, were recognized. Funding for research, lobbying, sponsoring, corporations, and brands were addressed as silent factors, because participants rarely talked about those elements during the interviews. For example, sponsoring, corporations, and brands were not mentioned in the data. COVID-19 was identified as a temporal element because it affected the OH over time. The WHO OH report and action plan, EU policies, and fluoride policies, which can be considered major issues, were frequently discussed during the interviews.

#### Social worlds/arenas map

The social worlds/arenas for improving the quality of dental care at the European and global policy levels are depicted in Figure 1. The 3 subarenas that lie within the overarching arena of QI in OH at the European and global policy on this map are networking, policy, guidance, and a wide variety of social worlds for organizations representing Europe and the countries of the globe within these contexts.

Policy organizations have the power to make decisions and contribute to laws and regulations. In contrast, organizations in the guidance arena contribute to recommendations or suggestions via standards or guidelines that provide best practices or preferred methods of achieving desired results. Organizations in the networking arena organize conferences, congresses, workshops, and seminars to bring together various stakeholder groups, member states, or countries. WHO, FDI, and IADR are at the center, influencing policy, providing guidance, and networking. The EU Commission is involved in networking and policy since it drives EU policies and uses networking to promote communication between member states. In contrast, the ICHOM, Council of European Dentists, and Lancet OH Commission contribute to the guidance and networking arena as they make the standards and guidelines for the national or regional level. Finally, the Global Child Dental Fund is located outside of these 3 arenas yet close to networking because it is a charitable organization that engages in networking.

#### Positional map

The positional map created in this study is based on interviews and lays out 4 different positions, as shown in Figure 2. This map depicted different positions within important discursive concerns arising in the data. The horizontal axis represents the efforts toward the QI in OH at the European and global policy level and ranges from low efforts (−) to high efforts (+). The vertical axis indicates the perception regarding the need for QI and ranges from low (−) to high (+) perception of need. The 4 recognized positions from the interviews are discussed below.

#### Position 1: QI is not a priority and underexamined

During the interviews, participants noted that QI in OH, in general, is an underexamined field. It has been put sidelined and not prioritized adequately due to other issues and needs. Since there is not much being done regarding QI, it has been assigned a low-effort position but a bit higher perception of need for QI.

Well, absolutely, 100%. I think it’s a completely under-examined area in dentistry. I mean, just speaking to my dental school completely under-examined area. And it’s the thing that kind of gets pushed aside when there are other issues that kind of raise to take priority. (INT_1, Pos. 22)

#### Position 2: Quality of OH needs to improve, although the contexts and needs of each country might vary

This position is high in the perception of need but low in effort. Almost all participants agreed that the quality of OH needs to be improved in Europe and worldwide despite variations in each country’s needs and context; that is why it took a higher position in perception of need for QI. In terms of efforts, it held a position between low and middle because a few actions and efforts have already been taken to improve the quality of OH.

Quality is an arbitrary term. So, the quality is very different in different parts of the world. (INT_4, Pos. 38)

#### Position 3: Prioritizing the outcome and quality measures

Prioritizing the outcome and quality measures took a high position in the perception of need regarding the QI because participants said these parameters are essential for QI in the dental setting. It placed a middle position for efforts, as some initiatives have been taken to measure the outcome and quality, such as setting a standard for outcome measures.

Measurements are an important element when we’re trying to improve the quality of care in general. (INT_7, Pos. 126)

#### Position 4: Dialogue between organizations is essential and is already happening

The final position of the map reflects a high perception of need and effort, as most participants agreed that interorganizational discussions are essential for enhancing the quality of OH policy at both the European and global levels. Also, it has been identified from the interviews that dialogues are already happening, and the organizations have made and achieved reasonable efforts.

There are big congresses all over Europe every year attracting people from all the neighbour countries (INT_3, Pos. 62)

It is significant to note that the results of our interviews did not yield a position that was very high in effort and low in perceived need, which is why this map area appears empty.

## Discussion

The study generated 4 main themes and 3 maps from the data collected through semi-structured interviews and desk research, which provided insights into the current understanding, needs, efforts, and actions in the context of European and global policy making for improving OH quality. Despite demonstrating a broad understanding of QI, participants mentioned barriers preventing impactful action and prioritization. The findings revealed that while a few efforts have been made toward QI in OH, there remains significant room for improvement through better policy making and stakeholder engagement. Furthermore, participants acknowledged the critical role collaborative stakeholder engagement can play in driving meaningful change in this area across Europe and globally.

The first theme was “perception and understanding of QI in OH policy making,” where participants showed wide understanding and emphasized evidence-based interventions and patient-centered attributes such as accessibility, experience, safety, timeliness, and quality of life as fundamental to optimal OH quality. This aligns with an existing international working definition for the quality of OH, which comprises 7 domains: patient safety, effectiveness, efficiency, patient centeredness, equitability, timeliness, and access to care (Righolt et al. 2020). In contrast, one study found that oral health policy makers have poorly understood and often overlooked oral health, which has led to a lack of urgency in promoting OH (Glick et al. 2023). The findings of this study and previous research indicated that for QI, it is essential to recognize the needs and contexts (Coles et al. 2020), learn from best practices, and share knowledge (Hughes 2008; NHS England 2022). Our findings revealed concerns regarding the absence of a Europe-wide framework and goals for improving OH quality, which is consistent with previous studies (“Platform Announces Recommendations for EU-Wide Action on Oral Health” 2015). Some participants were unaware of existing QI efforts, policies, and guidelines affecting OH at the international and European policy levels. A few individuals stated that they were not familiar with global and Europe-wide QI dialogues and activities. This could be because the efforts and actions taken are not sufficiently promoted. Thus, persons working in the fields of policy making, governance, and guidance are unaware of these efforts and initiatives. Studies in the past also showed that there is unawareness of OH among the international health community. Beaglehole and Greenspan indicated in the early 2000s that the wider international health community is generally unaware of the startling disparities within and across nations in the burden of oral disease worldwide and the availability of care (Greenspan 2007; Beaglehole et al. 2009). This sentiment is still visible as shown in our interviews, although awareness of the burden of OH disease has risen, and unawareness about the following steps to take action against the disease burden still exists.

The second theme involved the “prioritization of QI,” which recognizes a need to improve the quality of OH across Europe and globally. The existing literature emphasized the need for immediate enhancements in OH systems (Watt et al. 2019; Glick and Williams 2021). Our study findings highlighted insufficient support for QI and a lack of priority. According to the literature, worldwide OH suffers from a lack of political priority, particularly in low- and middle-income nations, due to complicated difficulties in the current global OH sector (O’Brien et al. 2022). Potential barriers and drivers of OH QI were discussed as shown in our results. This study found that having universal goals and duties, such as international standardization, UHC, international collaboration, networking, learning from others, monitoring, and outcome-oriented care, are some vital components for QI internationally, recognized by previous literature as well (Varkey et al. 2007; Hughes 2008; Apon et al. 2022). This result is also consistent with other literature that mentions UHC as an essential element that can help reduce inequality (Ghanbarzadegan et al. 2021). Lack of data and access to care were identified as vital barriers in our findings, also identified by past research. Winkelmann, Gómez Rossi, and van Ginneken said that almost every aspect of OH lacks sufficient data, making well-informed policy decisions difficult (Winkelmann et al. 2022). The challenges to working on QI were mentioned, such as leadership and governance issues and a lack of regulatory bodies. Governance was defined in the existing literature as crucial for improving political and financial support for OH, strengthening leadership, and forming mutually beneficial partnerships inside and beyond the health sector (Hughes 2008; Listl et al. 2023; WHO 2023).

Our third theme addressed the current “efforts and actions for the QI.” Despite the organisations efforts and actions to promote quality of OH, the results show that there is scope for improvement through policy making in Europe and globally. According to the findings, while some organizations have acted in this area, participants reported that other entities have been less active or consider existing initiatives insufficient. Previous research coincided with the findings of this study, suggesting actions to encourage global improvements in OH that focus on collaboration, sharing of knowledge, and policy development (Glick et al. 2023).

The final theme addressed “stakeholder engagement.” This study and the existing literature found the importance of stakeholder engagement and dialogue between organizational members. Involving various stakeholders in a broad advocacy group is crucial to increasing access to OH coverage and enhancing dental quality (Ticku et al. 2021). The findings of this study showed that an intersectoral approach should be employed to improve the quality of OH, and interprofessional collaboration was recognized as extremely important. Recent research argued that interprofessional and intersectoral collaboration and transdisciplinary learning could provide a roadmap for sustainable OH (Fisher et al. 2023).

The SitA is a method from the social sciences and has not yet been used very often in oral health research. In this study, the SitA has proven to be a suitable method to analyze the current status of QI in OH on a Europe-wide and global level. Through looking into various OH organizations and interviewing their members, we were able to create a combined table of the different elements seen in the situational map. This map gives a new perspective on elements that can influence QI in OH, as it does not look at the individual organizations but at their work and influence together. The social worlds/arenas map showed the arenas where the organization fields of activity overlap. This can be seen as an opportunity for dialogue and collaboration in the future. The positional map was created primarily on the basis of the discourses that we found relevant from the interviews, as these were the strong positions that were taken by the participants. The map showed that the need for QI in OH is relatively highly perceived. This suggests that the need for QI must first be recognized to then make efforts toward QI. The map shows that this step from perception to action has not yet been taken by the wider oral health community.

### Strengths and Limitations

This study’s primary strength lies in the fact that it is the first qualitative study, to our knowledge, to focus on key actors’ understanding, efforts, and actions in the European and global OH policy-making context for improving OH quality. This study stands out for including a diverse group of participants from 11 organizations, 7 international and 4 from Europe, with varying experience levels, offering a comprehensive grasp of the status quo of QI, requirements, initiatives, and actions in European and global OH policy making. Moreover, the participants generally had a strong desire to discuss the issue of this research, as seen by the length of the interviews, which frequently lasted longer than 45 min.

This study’s limitations must also be considered. The study has a sample size of 13 participants, which hinders the ability to make definitive conclusions for all high- and low-middle-income countries. Nevertheless, the participants represent highly influential organizations and are very experienced in the European and/or international OH policy-making and QI field, which is the subject area of this study. Furthermore, the qualitative study design does not aim to generalize the findings but to reach data saturation, which was achieved. In addition, the impact of the researchers background and perspectives on the analysis and interpretation of data in inductive qualitative research cannot be ignored.

## Conclusion

In summary, the SitA concluded that while there is a good understanding of QI in OH, barriers identified in this study can prevent effective action and prioritization. Our findings highlight the need for multiple strategies to address critical challenges. Key recommendations include promoting stronger partnerships between stakeholders to improve OH at European and global policy levels and addressing the identified barriers as well as the current state of OH policy to inform future policies and actions. Finally, identifying the gap between knowledge and action, this study emphasizes the critical need for participatory action research in OH policy making. A participatory approach, particularly in the context of the DELIVER project, can bridge this gap by engaging stakeholders in prioritizing resource allocation for more effective interventions.

### Author Contributions

S. Akter, contributed to design, data acquisition, analysis, and interpretation, drafted and critically revised the manuscript; V. Fehrer, contributed to design, data acquisition, analysis, and interpretation, critically revised the manuscript; M. Lorenz, contributed to conception, design, data acquisition, critically revised the manuscript; P. Jeurissen, S. Listl, contributed to conception, data acquisition, critically revised the manuscript. All authors gave final approval and agree to be accountable for all aspects of the work.
